# Involvement of Gut Microbiota in Schizophrenia and Treatment Resistance to Antipsychotics

**DOI:** 10.3390/biomedicines9080875

**Published:** 2021-07-23

**Authors:** Mirko Manchia, Andrea Fontana, Concetta Panebianco, Pasquale Paribello, Carlo Arzedi, Eleonora Cossu, Mario Garzilli, Maria Antonietta Montis, Andrea Mura, Claudia Pisanu, Donatella Congiu, Massimiliano Copetti, Federica Pinna, Valerio Pazienza, Alessio Squassina, Bernardo Carpiniello

**Affiliations:** 1 Unit of Psychiatry, Department of Medical Sciences and Public Health, University of Cagliari, 09127 Cagliari, Italy; mirkomanchia@unica.it (M.M.); pasqualeparibello@gmail.com (P.P.); carloarzedi@yahoo.it (C.A.); cossu.e90@gmail.com (E.C.); m.garzi@gmail.com (M.G.); mary.montis@tiscali.it (M.A.M.); andremura88@gmail.com (A.M.); fedepinna@inwind.it (F.P.); bcarpini@iol.it (B.C.); 2 Unit of Clinical Psychiatry, University Hospital Agency of Cagliari, 09127 Cagliari, Italy; 3 Department of Pharmacology, Dalhousie University, Halifax, NS B3H4R2, Canada; 4 Unit of Biostatistics, Fondazione IRCCS Casa Sollievo della Sofferenza Hospital, 71013 San Giovanni Rotondo, Italy; a.fontana@operapadrepio.it (A.F.); m.copetti@operapadrepio.it (M.C.); 5 Division of Gastroenterology, Fondazione IRCCS Casa Sollievo della Sofferenza Hospital, 71013 San Giovanni Rotondo, Italy; panebianco.c@gmail.com; 6 Unit of Neuroscience and Clinical Pharmacology, Department of Biomedical Sciences, Section of Neuroscience and Clinical Pharmacology, University of Cagliari, 09042 Cagliari, Italy; claudia.pisanu@unica.it (C.P.); dcongiu@unica.it (D.C.)

**Keywords:** severe mental disorders, psychosis, pharmacogenetics, pharmacogenomics, microbiome, diet, typical antipsychotics, atypical antipsychotics

## Abstract

The gut microbiota is constituted by more than 40,000 bacterial species involved in key processes including high order brain functions. Altered composition of gut microbiota has been implicated in psychiatric disorders and in modulating the efficacy and safety of psychotropic medications. In this work we characterized the composition of the gut microbiota in 38 patients with schizophrenia (SCZ) and 20 healthy controls (HC), and tested if SCZ patients with different response to antipsychotics (18 patients with treatment resistant schizophrenia (TRS), and 20 responders (R)) had specific patterns of gut microbiota composition associated with different response to antipsychotics. Moreover, we also tested if patients treated with typical antipsychotics (n = 20) presented significant differences when compared to patients treated with atypical antipsychotics (n = 31). Our findings showed the presence of distinct composition of gut microbiota in SCZ versus HC, with several bacteria at the different taxonomic levels only present in either one group or the other. Similar findings were observed also depending on treatment response and exposure to diverse classes of antipsychotics. Our results suggest that composition of gut microbiota could constitute a biosignatures of SCZ and TRS.

## 1. Introduction

Schizophrenia (SCZ) is a devastating mental disorder characterized by severe alterations in thought process, affectivity, perception and social cognition [1]. It has been estimated that 21 million people are affected by schizophrenia worldwide [2], with a prevalence ranging from 0.33% to 0.75% across studies [3,4]. Schizophrenia is one of the top 15 leading causes of disability worldwide [5], and is associated with premature mortality and high rates of medical comorbidities [6]. Although there are still uncertainties on the precise pathophysiological underpinnings of SCZ, the hypothesis of altered neurodevelopmental processes has been largely discussed and supported [7]. Since its postulation almost 35 years ago [8], this hypothesis has been often revised and refined, but there is consensus that the disrupted development of the central nervous system (CNS) in SCZ can be determined by the interplay of several determinants of risk, including, among others, genetic predisposition, prenatal exposure to infections, season of birth and childhood and adulthood adversities [9]. 

Clinical, neuroimaging and genomic data have partly shed light on the neurobiological underpinnings of SCZ, but these advancements have not translated into effective preventative strategies or pharmacological treatments for all the individuals affected by SCZ. Indeed, there is a large proportion of patients with suboptimal responses to pharmacological treatments and clear unmet needs in the management of those clusters of psychopathological manifestations (i.e., negative and cognitive symptoms) that most prominently influence the functioning of affected individuals and impede clinical recovery. In addition, about one-third of patients with SCZ show poor response to antipsychotic treatments and is eventually diagnosed as treatment resistant [10,11]. Treatment resistance (TR) to antipsychotics is commonly defined as the persistence of symptoms despite two or more trials of antipsychotics of adequate dose and duration with documented adherence [12,13]. Clozapine, an atypical antipsychotic, is the only treatment currently indicated for treatment resistant SCZ (TRS) [14,15]. Nevertheless, treatment with clozapine presents a number of limitations and barriers that significantly limit its use, including potentially severe side effects, mandatory blood testing, reduced adherence and difficulty in identifying suitable patients [16]. Moreover, clozapine is ineffective in approximately 20% of TRS patients who are defined as “ultra-resistant” [15,17]. The search for clinical and biological predictors of TRS has been so far hampered by the heterogeneity of the phenotypic definition and a lack of specific clinical signatures predicting its manifestation. However, several studies suggest that TRS differs from treatment responsive SCZ for different features, such as functioning of the dopaminergic, glutamatergic and serotonergic pathways, regulation of the immune system, and white matter abnormalities among others [18,19,20,21]. In general, however, response to medications, including antipsychotics, remains conditioned by a large set of pharmacokinetics and pharmacodynamics characteristics, and several of these processes can be significantly influenced by the activity of microorganism in the intestine [22]. These bacteria constitute the gut microbiota, a large population comprising 40,000 bacterial species and 1800 phyla involved in key processes important to maintain body homeostasis [23]. Recent studies have shown that several bacteria species encode enzymes capable of metabolizing most of the drugs commonly prescribed in humans, including antipsychotics, and that this activity can metabolize extensively diverse drugs, possibly reducing their efficacy [22]. Interestingly, drug-microbiota interactions vary significantly between individuals suggesting that the study of gut microbiota might have potential applications in personalized medicine [24]. The involvement of gut microbiota in SCZ has been hypothesized and explored in both preclinical and clinical studies. Findings suggest that patients with SCZ present important differences in the composition of gut microbiota compared to healthy controls [25], and that response to antipsychotics might be influenced by gut microbiota [26]. Moreover, clinical trials with probiotics or prebiotics have provided some support to their efficacy in ameliorating some of the symptoms of SCZ [27]. Furthermore, it has been shown that fecal transplantation from drug-free patients with SCZ causes SCZ-like abnormal behaviors and dysregulated kynurenine metabolism in mice [28]. Findings suggesting a role of the gut microbiota in SCZ contribute to support the hypothesis of the implications of the gut–brain axis in mental disorders [29]. This axis concerns the complex bi-directional signaling system between the brain and the gut, which constitutes a communication network including the CNS, the spinal cord, the autonomic nervous system, the enteric nervous system, the immune system and the hypothalamic pituitary adrenal axis [30,31]. Indeed, the microbiota produces molecules that act at distal sites, thus mimicking the functions of an endocrine organ, and is involved in modulating high-order brain functions. The composition of the gut microbiota is highly influenced by environmental factors (diet, physical activity, smoke, substance abuse, etc.) and by acute and chronic disorders not limited to the gastrointestinal tract [32,33]. This composition also changes physiologically during the development of the human body, and some of these changes overlap with sensitive periods in the neurodevelopment [34]. It has been hypothesized that modifiers impacting on these changes in the sensitive periods (critical windows) might affect the correct development of the CNS through the gut–brain axis. Specifically, altered or disrupted gut microbiota in prenatal periods, at weaning and/or in adolescence might represent risk factors for neurodevelopmental disorders, including SCZ [34]. While this hypothesis has been difficult to explore due to the complex study design required to investigate it adequately, it provides a fascinating insight on the biological underpinnings of SCZ. 

In this context, we carried out a study in which we characterized the composition of the gut microbiota in patients affected by SCZ with the threefold aim of comparing gut microbiota composition: (1) between patients affected by SCZ and healthy controls (HC); (2) between TRS and responsive patients, as well as between TRS and HC; and (3) between patients affected by SCZ treated with typical (T) (first-generation) and atypical (AT) (second generation, including aripiprazole) antipsychotics. 

## 2. Materials and Methods

### 2.1. Study Sample 

The recruitment process of our cohorts of 38 patients affected by SCZ and 20 HC took place at the community mental health center of the Section of Psychiatry of the Department of Medical Science and Public Health, University of Cagliari and University Hospital Agency of Cagliari and was previously detailed [35]. The study was conducted according to the guidelines of the Declaration of Helsinki, in compliance with the Italian national legislation, and approved by the Ethics Committee of the University Hospital Agency of Cagliari (PG/2018/11693, 5th of September 2018). Briefly, we included patients affected by SCZ diagnosed according to DSM IV-TR criteria [36], who: (a) were able to express a consent to participate formulated by signing the consent form, (b) were of age between 18 and 70 years-old, and (c) had at least 6 months of stability before recruitment. Recruited subjects were assessed by trained mental-health professionals (psychiatry residents or senior clinical staff). Clinical information was collected through direct interview of the patient as well as through a systematic assessment of existing medical records. Whenever possible we collected collateral information from at least one first degree relative or significant other, after obtaining the consent from the participant. Treatment resistance was defined according to the criteria of Kane et al. [14], and is based on the clinical course and evaluation of treatment response patterns. In total, 18 patients were TRS, and 20 were responders. As for the class of antipsychotics, 20 patients were treated with typical and 31 with atypical antipsychotics. We applied the following exclusion criteria: (1) presence of acute infections; (2) presence of chronic autoimmune inflammatory conditions (e.g., rheumatoid arthritis, thyroiditis); (3) presence of eating disorders; (4) presence of post-traumatic stress disorder; (5) presence of current substance use disorders; (6) presence of neurological disorders; (7) past traumatic brain injury; (8) presence of severe co-morbidities that may influence molecular testing (such as cancer, HIV infection). The inclusion criteria for HC comprised: (1) the absence of a personal history of mental disorders, (2) the willingness to participate in the study, (3) absence of acute infections; (4) absence of chronic autoimmune inflammatory conditions (e.g., rheumatoid arthritis, thyroiditis); (5) absence of past traumatic brain injury; (6) absence of severe co-morbidities that may influence molecular testing (such as cancer, HIV infection). In addition, in both SCZ and HC we excluded individuals that used antibiotics in the 3 months preceding the sampling procedure or had a chronic use of probiotics. 

### 2.2. Sample Collection and DNA Extraction

Each study participant provided a fresh stool sample in a tube containing a DNA stabilization buffer (Canvax Biotech, Cordoba, Spain Cat N ° SC0012), from which DNA was extracted. following the manufacturer’s instructions of the QIAamp DNA Stool Mini Kit (Qiagen, Milan, Italy, Cat N ° 51504), according to the protocol optimized to increase the ratio of nonhuman DNA to human DNA. In detail, lysis was performed by adding 1.4 mL Buffer ASL (proprietary buffer for lysis) to 250 µL of faecal sample and vortexing to obtain a thorough homogenization. Then, the faecal suspension was heated for 5 min at 90 °C to promote the lysis of cells difficult to dissolve (i.e., Gram-positive bacteria). At the end of the isolation protocol, DNA was checked for concentration and purity and stored at −30 °C until use.

### 2.3. Next-Generation Sequencing of Bacterial 16S rRNA Gene

In total, 10 ng of each fecal DNA underwent library preparation for 16S rRNA gene amplicon sequencing on an Illumina MiSeq device, as previously described [37]. Briefly, the V3-V4 region was amplified using KAPA HiFi HotStart ReadyMix (Roche Diagnostics, Milan, Italy, Cat N ° 07958935001), samples were barcoded with Nextera XT Index Kit (Illumina, Milan, Italy, Cat N ° FC-131-1002), libraries were pooled in equimolar concentrations and subjected to 2 × 300 paired-end sequencing, using the MiSeq Reagent Kit v3 (600 cycle) (Illumina, Milan, Italy, Cat N ° MS-102-3003).

### 2.4. Bioinformatic Analysis

The de-multiplexed reads generated by MiSeq were processed using the 16S Metagenomics GAIA v.2.0 software (http://www.metagenomics.cloud, Sequentia Biotech 2017, access date: 4 October 2019; Benchmark of GAIA 2.0 using published datasets available online at: http://gaia.sequentiabiotech.com/benchmark, access date: 4 October 2019), as described in Fontana et al. [38]. Read pairs were quality-controlled (i.e., trimming, clipping and adapter removal) based on FastQC and BBDuk and mapped with BWA-MEM against the custom databases (based on NCBI), to obtain the taxonomic profile of each sample.

### 2.5. Statistical Analysis

Clinical characteristics of patients with SCZ and HC were reported as median along with interquartile range (i.e., first-third quartiles) and observed frequencies (and percentages) for continuous and categorical variables, respectively. For each continuous variable, the assumption of normality distribution was checked by means of quantile–quantile (Q-Q) plots and Shapiro–Wilks test. In the presence of non-normal distributions, comparisons between groups were performed by Mann–Whitney U test (or Kruskal–Wallis test as appropriate) and χ^2^ test (or Fisher exact test, as appropriate) for continuous and categorical variables, respectively. Stacked bar charts were used to show the gut microbiota composition (i.e., mean relative abundance %) at phylum, family, genus and species levels between SCZ and HC. We applied the Penalized Logistic Regression Analysis (PELORA) algorithm, to identify panels of bacterial populations that best discriminated groups (i.e., SCZ versus HC or comparisons among SZ subgroups according to presence/absence of TR to antipsychotics) [39]. To this purpose, the relative abundance (%) of each bacterium was first logistic transformed (i.e., by calculating the natural logarithm of the ratio between the relative abundance proportion and its complimentary) and then standardized (computing a Z-score) by subtracting its mean and dividing by its standard deviation (SD). Both mean and SD were computed in the sample which included all the subjects involved in the comparison. When the relative abundance was exactly 0%, the logistic transformation cannot be performed for that value and, to overcome this issue, such percentage was replaced by 0.001% for the computation of Z-score only. Once a pattern was identified, its centroid was computed by the mean of the Z-scores of the involved bacteria. To calculate centroids, Z-scores of some bacteria could be sign-flipped (reversed) to put their values in the same direction suggested by the centroid. PELORA algorithm was also set to accommodate clinical variables: when a new predictor is added to the model, this can either be a group centroid or a clinical variable, depending on which yields better predictive value [39]. In details, when comparing patients with SCZ versus HC, penalized logistic models which included the centroid as predictor were adjusted for the effect of age at the sample collection, gender and body mass index (BMI) whereas, when comparing subgroups of patients with SCZ, models were adjusted for the effect of age at SCZ onset, illness duration, gender, BMI, treatment duration and the presence of concomitant drugs. Moreover, when comparing patients with SCZ versus HC, covariates related to lifestyle (i.e., diet, smoke and drink habits, presence of physical activity) were not considered because they were intrinsically related to the HC profile. In accordance with the analysis protocol, two different free parameters were set in the PELORA algorithm: the number of centroids and the penalty parameter (λ). The number of centroids was set to 1, because we were mainly interested to detect only one informative pathway for each scenario whereas several different combinations of λ = (0, 1/32, 1/16, 1/8, 1/4, 1/2, 1) were evaluated, performing 200 bootstrap resampling of data and recording the overall misclassification rate. For each specific scenario, the penalty parameter that achieved the lowest median misclassification rate (across the bootstrap samples) was chosen. Comparisons between Z-scores were performed using two-sample *t*-test. Heatmaps of normalized Z-scores (from 0 to 1) of relative abundances of bacterial populations identified by PELORA algorithm along with the corresponding centroid and boxplots of centroid Z-scores were created. Two-sided *p* < 0.05 was set as statistical significance threshold. All statistical analyses and plots were performed by the computing environment R (packages: supclust, ggplot2, gridExtra) [40].

## 3. Results

### 3.1. Sample Characteristics

The SCZ patients enrolled in the study were classified in two subgroups based on their pattern of response to antipsychotics, namely TRS or responsive (R). Clinical and demographic characteristics of these subgroups of SCZ patients as well as of HC are summarized in Table 1. These three groups were homogeneous for all the examined characteristics except for BMI (*p* < 0.001), smoking habits (*p* = 0.021), drinking habits (*p* = 0.003) and for physical activity (*p* = 0.012).

### 3.2. Comparison of Gut Microbiota Composition between Patients Affected by SCZ and HC

Next-generation sequencing analysis produced on average 128,568 (±113,337) quality-filtered read pairs for each of the 58 study participants (38 patients with SCZ and 20 HC). 

**Figure 1:**
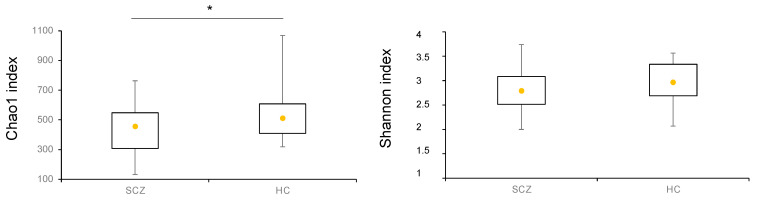
Boxplots of Chao1 index (richness) and Shannon index (alpha-diversity) calculated at the genus level in all patients affected by SCZ and HC. Yellow dot in each plot represents the median value. * *p*-value < 0.05.

The richness metric Chao1 and the alpha-diversity index Shannon were assessed at the genus level in SCZ and HC, as represented in Figure 1. While no change was observed as for alpha-diversity, a statistically significant decrease in richness emerged in SCZ compared to HC. 

**Figure 2:**
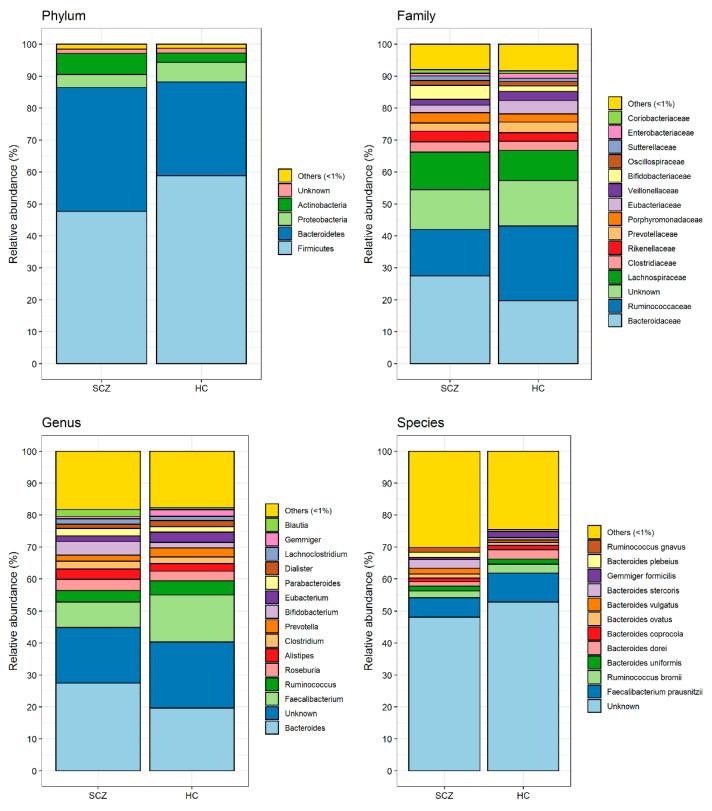
Gut microbiota composition (i.e., mean relative abundance %) at phylum, family, genus and species levels grouped by patients affected by SCZ and HC.

**Figure 3:**
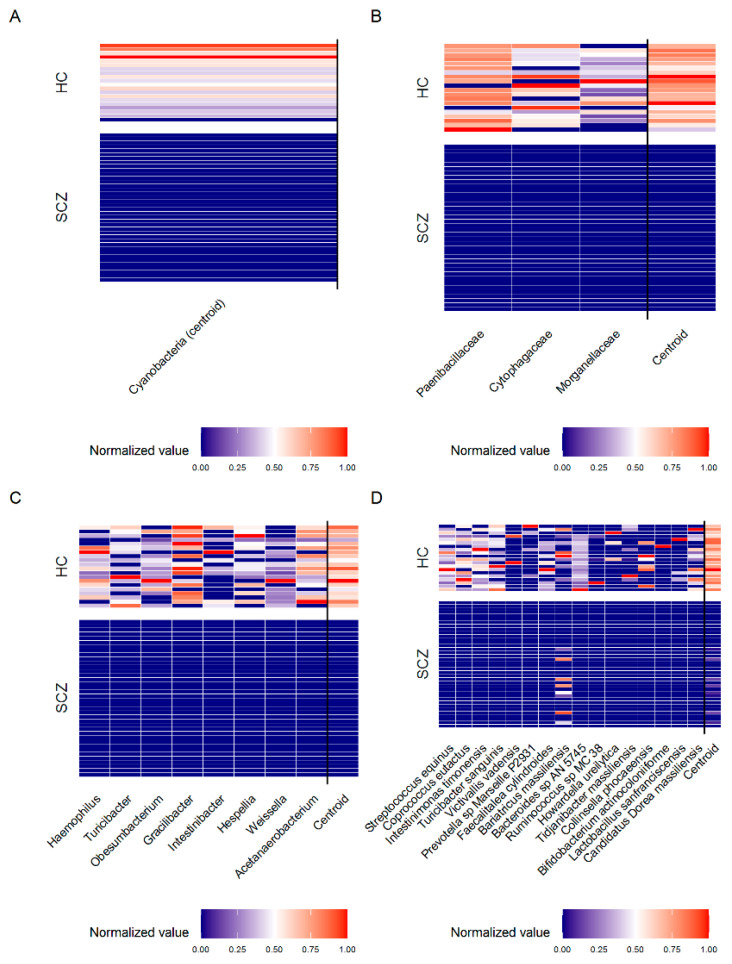
Heatmaps of standardized relative abundance of bacterial populations (normalized Z-scores) identified by the penalized logistic regression analysis algorithm, at phylum (**A**), family (**B**), genus (**C**) and species (**D**) levels grouped by patients affected by SCZ and HC.

**Figure 4:**
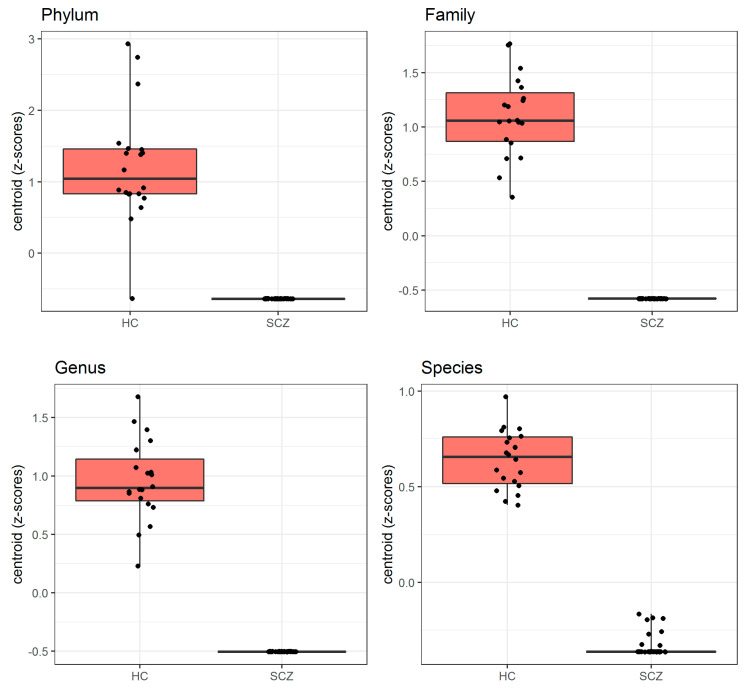
Boxplots of centroid Z-scores, computed by the penalized logistic regression analysis algorithm, which discriminated all patients affected by SCZ from HC.

Figure 2 shows the mean relative abundance of gut microorganisms detected at the phylum, family, genus and species level in SCZ and HC, respectively. Based on these data, we applied the PELORA algorithm to find out microbial patterns discriminating all patients affected by SCZ from HC. As reported in Table 2, and graphically represented by the heatmaps in Figure 3, several bacteria at the different taxonomic levels were detected in HC but were missing in patients with SCZ. In detail, the phylum *Cyanobacteria*, the families *Paenibacillaceae*, *Cytophagaceae* and *Morganellaceae*, the genera *Acetanaerobacterium*, *Haemophilus*, *Turicibacter*, *Obesumbacterium*, *Gracilibacter*, *Intestinibacter*, *Hespellia* and *Weissella*, and the species *Streptococcus equinus*, *Coprococcus eutactus*, *Turicibacter sanguinis*, *Victivallis vadensis*, *Prevotella *sp*. Marseille P2931*, *Faecalitalea cylindroides*, *Intestinimonas timonensis*, *Bacteroides *sp*. AN 5745*, *Collinsella phocaeensis*, *Candidatus Dorea massiliensis*, *Ruminococcus *sp*. MC 38*, *Bifidobacterium actinocoloniiforme*, *Tidjanibacter massiliensis*, *Howardella ureilytica* and *Lactobacillus sanfranciscensis* only populated the gut of HC, being absent in SCZ subject. Figure 4 represents the distribution of centroid z-scores computed by PELORA in patients with SCZ and HC, showing a high discriminatory power for the identified bacterial patterns at each taxonomic level considered.

### 3.3. Comparison of Gut Microbiota Composition between TRS and R Patients and between TRS and HC, R and HC

As reported in Table 3, PELORA identified patterns of bacteria characterizing solely the gut microbiota of TRS or R patients with SCZ. Specifically, the phyla *Candidatus Saccharibacteria* and *Tenericutes*, and the genera *Actynomyces* and *Porphyromonas* were found in TRS but not in R patients, whereas the families *Flavobacteriaceaea* and *Enterococcaceae*, and the species *Flintibacter butyricus* were absent in TRS but represented in R group. In addition, we sought to compare microbiota composition of either R and TRS patients with HC. As shown in Table 4, *Candidatus Saccharibacteria*, *Cyanobacteria* and *Tenericutes* at the phylum level, were found to populate the gut of HC but not of R SCZ patients. Conversely, *Flavobacteriaceae* and *Desulfobacteraceae* at the family level, *Fenollaria*, *Mitsuokella*, *Harryflintia* and *Mailhella* among the genera, and *Flintibacter butyricus* at the species level, were solely found in R patients while they were absent in HC. Table 5 reports bacterial patterns discriminating TRS patients from HC, as calculated by PELORA. In this comparative analysis, the phylum *Cyanobacteria*, the families *Enterococcaceae*, *Paenibacillaceae*, *Cytophagaceae*, *Hafniaceae* and *Pasteurellaceae*, and the genera *Murimonas*, *Haemophilus*, *Peptococcus*, *Weissella*, *Enterobacter*, *Hespellia* and *Turicibacter* were all measured in HC but absent in TRS patients, while the genera *Fusicatenibacter* and *Eggerthella* were enriched in HC with respect to TRS patients. At the species level, instead, *Bacteroides *sp*. Marseille P3108*, *Pseudoflavonifractor capillosus*, *Erysipelatoclostridium ramosum*, *Papillibacter cinnamivorans* and *Clostridium *sp*. BPY5*, all found in TRS patients but not in HC, represented the pattern best discriminating these two groups.

### 3.4. Comparison of Gut Microbiota Composition between SCZ Patients Treated with Typical and Atypical Antipsychotics

When comparing the gut microbiota of SCZ patients treated with T and AT antipsychotics with the PELORA algorithm, a remarkable pattern of taxa discriminating the two groups was observed, as listed in Table 6. At the phylum level, these two groups were best discriminated by *Bacteroidetes*, *Fusobacteria* (more represented in T group) and *Tenericutes*. Among bacterial families, *Fusobacteriaceae*, *Streptomycetaceae*, *Helicobacteraceae*, *Bacillaceae*, *Oxalobacteraceae* and *Erysipelotrichaceae* were more abundant in T SCZ patients, the opposite was for *Synergistaceae*, *Veillonellaceae* and *Clostridiales Family XIII Incertae Sedis*, whereas *Clostridiales Family XII Incertae Sedis* were only found in AT but were absent in T group. *Fusobacterium*, *Butyricimonas*, *Blautia*, *Paraprevotella*, *Klebsiella* (all more abundant in T group), together with *Olsenella* and *Cronobacter* represented the genus pattern best discriminating the microbiota between these two classes of treatment. Finally, at the species level, *Parabacteroides merdae*, *Ruminococcus *sp*. AT10*, *Clostridium piliforme*, *Enorma massiliensis*, *Megamonas rupellensis*, *Bacteroides ovatus*, *Butyricimonas *sp*. GD2* and *Ruminococcus *sp*. DJF VR70k1* were more represented in T SCZ patients, together with *Odoribacter *sp*. S90*, which was completely absent in AT SCZ subjects. Conversely, *Faecalibacterium prausnitzii* and *Clostridium aldenense* were increased in AT SCZ patients, together with *Tetragenococcus halophilus* which was not detected in T SCZ individuals.

## 4. Discussion

We used a cross-sectional, case-control design to test the hypothesis that patients affected by SCZ might have a gut microbiota composition significantly different from that of HC, and that these differences could be even more specific in SCZ patients depending on the pattern of clinical response to antipsychotics (TR versus R) or the type of pharmacological class (T versus AT). We found that, in line with a substantial body of the literature [25], the diversity in the composition of the gut microbiota of patients affected by SCZ was significantly reduced compared to HC. Furthermore, the penalized logistic regression analysis found that: (a) many microorganisms were detected in HC but absent in SCZ patients, namely those of the genus *Acetanaerobacterium*, *Haemophilus, Turicibacter*, *Gracilibacter*, *Obesumbacterium, Intestinibacter, Hespellia* and *Weissella*; (b) compared to R patients affected by SCZ, the gut microbiota of TRS had an overrepresentation of the phyla *Candidatus Saccharibacteria* and *Tenericutes*, and the genera *Actynomyces* and *Porphyromonas.* Conversely, the families *Flavobacteriaceaea* and *Enterococcaceae*, and the species *Flintibacter butyricus* were absent in TRS but represented in R group. Furthermore, a specific composition of the gut microbiota distinguished TRS and R patients form HC. Specifically, the species *Bacteroides *sp*. Marseille P3108*, *Pseudoflavonifractor capillosus*, *Erysipelatoclostridium ramosum*, *Papillibacter cinnamivorans* and *Clostridium *sp*. BPY5* were present in TRS patients, and *Flintibacter butyricus* in the R subgroup, but not in HC; and (c) several species, including *Parabacteroides merdae*, *Ruminococcus *sp*. AT10*, *Clostridium piliforme*, *Enorma massiliensis*, *Megamonas rupellensis*, *Bacteroides ovatus*, *Butyricimonas *sp*. GD2*, *Ruminococcus *sp*. DJF VR70k1* and *Odoribacter *sp*. S90* were more represented in SCZ patients treated with T antipsychotics compared to those treated with AT. Conversely, *Faecalibacterium prausnitzii*, *Clostridium aldenense* and *Tetragenococcus halophilus* were increased in AT treated SCZ patients compared to those treated with T antipsychotics. Taken together, these results suggest the presence of distinct patterns of gut microbiota composition depending on the illness status, treatment response patterns and exposure to a diverse class of antipsychotic. Each of this set of findings has potential neurobiological relevance, and inherent limitations, and deserves to be commented on. 

The presence of a distinct signature of gut microbiota components in patients affected by SCZ compared to HC is consistent with emerging evidence that molecular substrates of social, cognitive and emotional domains, known to be altered in SCZ, might be influenced by life-long microbiota–gut–brain axis signaling [41]. Indeed, a recent study showed that the higher relative abundance of genus *Roseburia* in SCZ patients, compared to HC, was negatively correlated with the regional homogeneity indexes of brain regions putatively involved in the pathophysiology of SCZ, such as the right superior temporal cortex, the left cuneus and the right medial temporal cortex [42]. 

Our study highlighted a different representation of several types of bacterial genus and species in the gut microbiota of patients affected by SCZ compared to HC. Specifically, this statistically significant underrepresentation involved several species, i.e., *Coprococcus eutactus*, *Bariatricus massiliensis*, *Ruminococcus* sp. MC38 belonging to the *Clostridiales* order. *Clostridiales* are known to degrade branched chain amino acids (BCAAs), which share brain transporters with tryptophan. It is plausible that *Clostridiales* depletion, by increasing circulating concentrations of BCAA, could decrease brain tryptophan uptake and serotonin production, thus impacting on psychopathological dimensions of SCZ [43]. The gut microbiota of patients affected by SCZ was also lacking bacteria of the genus *Haemophilus*, which were overrepresented in HC. This finding is consistent with previous observations that found this specific signature in patients affected by SCZ, who, similarly to those studied in our study, had an illness duration longer than 10 years [44]. Other observations derived from our analysis should be considered in the context of the neurodevelopmental hypothesis of SCZ, namely the decreased relative abundance of *Intestinibacter* and *Weisella* in SCZ compared to HC. Both sets of finding appear at odds with the observations of increased levels of *Intestinibacter* in children with neurodevelopmental disorders, including autism [45], a disorder that shares some clinical features of SCZ, and of *Weisella* in infants born to mothers exposed to interpersonal violence compared to those with no/low exposure [46]. 

Our study also showed a distinct signature of gut microbiota composition in patients affected by SCZ who had a clinical history of response or resistance to antipsychotic treatments. Comparison with previous evidence is complicated by the lack of data examining gut microbiota composition in TRS patients. Nevertheless, we observed an enrichment of the genus *Actinomyces* and *Porphyromonas* in the gut microbiota of TRS patients compared to those responsive to antipsychotics. These bacteria, particularly the *Porphyromonas*, exert pro-inflammatory actions at the level of the intestine, increasing the permeability of the gut blood barrier, and creating a substantial dysbiosis [47]. Consistently, this genus was found overrepresented in the gut microbiota of individuals assuming methamphetamine, a compound known for increasing the systemic inflammatory status [48]. Conversely, the gut microbiota of treatment responsive patients with SCZ, compared to TRS, was populated by the *Flintibacter butyricus*, which is an important butyrate producer and could counteract the hyper inflammatory status, which plays a major role in the pathophysiology of SCZ [49]. Furthermore, the comparison between TRS patients and HC highlighted the overrepresentation of a number of species including *Papillibacter cinnamivorans* and *Erysipelatoclostridium ramosum* in the former group. *Papillibacter cinnamivorans* has been found increased in the gut microbiota of individuals affected by Parkinson’s disease [50], whose neurobiological underpinnings lie in the degeneration of dopaminergic neurons in the CNS, a neurochemical pathway known to be altered in SCZ. Of note, this increase of *Papillibacter cinnamivorans* appears to trigger local inflammation which might contribute to neurodegeneration, a phenomenon observable also in SCZ [50]. Interestingly, *Erysipelatoclostridium ramosum* has been shown to be involved in the synthesis of serotonin in the intestine [51], with a plausible link with the development of obesity but possibly also in creating an imbalance in the neurochemical signaling of this pathway relevant for SCZ by limiting the availability of L-tryptophan [52]. Finally, the comparison between patients with responsiveness to antipsychotics and HC showed a statistically significant overrepresentation of *Flintibacter butyricus* in the former group, which was seen also in the comparison with TRS patients, and that could be explained as a compensatory overexpression to counteract the hyperinflammatory state of SCZ.

The third set of comparison showed that patients treated with different classes of antipsychotics have distinct signatures of gut microbiota. Specifically, a number of butyrate producing bacteria, including *Erysipelotrichaceae*, *Butyricimonas*, *Blautia* and *Paraprevotella*, were increased in the group of T versus AT patients. However, the gut microbiota of the latter group was strongly enriched in another butyrate producer, *Faecalibacterium prausnitzii*, with potential anti-inflammatory effects. It should be noted however that the gut microbiota of the T patients subgroup beside beneficial bacteria expressed also potential pathogens such as *Fusobacteraceae*, *Helicobacteraceae* and *Klebsiella*, all linked to dysbiosis and increased levels of systemic inflammation.

Several limitations should be considered in the interpretation of our findings. First, our phenotypic data are cross-sectional and retrospective in nature, and this limits the ability of establishing causality (i.e., whether variation in the composition of the gut microbiota resulted from or preceded the onset of SCZ, or the start of specific antipsychotic treatments). It should be noted, however, that the characterization of the clinical response to antipsychotics was based on accurate information collected through longitudinal prospective follow-ups in some instances with lengths of decades, decreasing the risk of recall bias. A second, partially related to the first, limitation lies in the use of antipsychotic treatments that might have influenced the composition of the gut microbiota. It should be noted however, that the case-control comparison included the presence of drug treatment as covariate. Third, although information on diet was accurately collected at the time of stool specimen sampling, it is not possible to establish reliably how consistent were dietary habits among our patients. Fourth, the relatively small sample size could have impacted on the statistical power leading to a decreased sensitivity and specificity of our findings making our study a proof of concept. Of note, however, TRS is a relatively complex phenotype to characterize given its population prevalence (circa one-third of treated patients affected by SCZ), and the small evidence available in the literature has been gathered in samples of comparable size. Although exploratory in nature, our findings enrich the literature on the presence of distinct alterations of gut microbiota composition in SCZ compared to unaffected individuals, and point to specific biosignatures of TRS, either in comparison to R or to HC. These observations need to be validated in prospective assessments of drug-naïve patients with first psychotic episodes, ideally with faecal specimens sampled before the initiation of a trial with an antipsychotic and at regular intervals of time during the continuation of the treatment. This could permit to ascertain whether changes in the composition of the gut microbiota correspond to major psychopathological modifications, establishing causality. While the analysis of drug-naïve patients would be particularly difficult in the case of TRS, given that this tends to be a mid/long term outcome in the clinical course of SCZ, and patients with TRS have, by definition, been exposed to several trials of antipsychotics, such an approach could be feasible only using establishing prospective cohorts followed in the long-term, as for high-risk studies [53,54]. Finally, translational approaches, including those using faecal transplant of the gut microbiota from more severe SCZ cases, such as TRS, into germ free animals, could lead to the identification of specific phenotypic alterations in animal models, and eventually of biosignature which can be reverse translated and validated in humans.

## 5. Conclusions

In conclusion, even considering these limitations, our study suggested distinct signatures associated with the illness status, the pattern of treatment response and the exposure to a specific class of antipsychotics. These findings warrant replications in independent samples and/or inclusion in future quantitative synthesis to validate these signatures, identify plausible neurobiological links and design specific interventions that can reduce the identified dysbiosis.

## Figures and Tables

**Table 1:** Demographic and clinical characteristics of patients with SCZ classified according to their pattern of treatment response (TRS and R) and HC.

Variable	Category	TRS (*N* = 18)	R (*N* = 20)	HC (*N* = 20)	*p*-Value
Age (years)	Median [IQR]	44.0 [41.6–49.8]	50.0 [40.3–60.9]	37.7 [30.6–58.0]	0.277 *
Gender—N(%)	Males	16 (88.9)	18 (90.0)	13 (65.0)	0.102 ^#^
	Females	2 (11.1)	2 (10.0)	7 (35.0)	
BMI (Kg/m^2^)	Median [IQR]	27.3 [25.5–29.2]	26.9 [25.5–28.5]	22.7 [21.2–23.8]	<0.001 *
Family history for mental disorders—N(%)	No	9 (50.0)	11 (55.0)	11 (68.8)	0.542 ^#^
	Yes	9 (50.0)	9 (45.0)	5 (31.2)	
Diet—N(%)	Mediterranean only	10 (58.8)	13 (65.0)	18 (90.0)	0.445 ^#^
	Carbohydrates only	1 (5.9)	0 (0.0)	0 (0.0)	
	Vegetarian/Vegan only	1 (5.9)	0 (0.0)	0 (0.0)	
	Mediterran + iperproteic	1 (5.9)	2 (10.0)	0 (0.0)	
	Mediterran + ipercaloric	0 (0.0)	0 (0.0)	1 (5.0)	
	Mediterran + carbohydrates	2 (11.8)	1 (5.0)	1 (5.0)	
	Mediterran + iperproteic + carbohydrates	1 (5.9)	1 (5.0)	0 (0.0)	
	Mediterran + ipercaloric + carbohydrates	0 (0.0)	1 (5.0)	0 (0.0)	
	Iperproteic + carbohydrates	1 (5.9)	2 (10.0)	0 (0.0)	
Smoking habits—N(%)	Non-smoker	5 (27.8)	4 (20.0)	13 (65.0)	0.021 ^#^
	Smoker	11 (61.1)	11 (55.0)	4 (20.0)	
	Ex-smoker	2 (11.1)	5 (25.0)	3 (15.0)	
Drink habits—N(%)	None	12 (66.7)	13 (65.0)	3 (15.8)	0.003 ^#^
	One occasional drink	6 (33.3)	6 (30.0)	12 (63.2)	
	1-2 drinks per day	0 (0.0)	1 (5.0)	4 (21.1)	
Physical activity—N(%)	No	11 (61.1)	14 (70.0)	5 (25.0)	0.012 ^#^
	Yes	7 (38.9)	6 (30.0)	15 (75.0)	
Cardiometabolic comorbidities—N(%)	No	11 (61.1)	13 (65.0)	16 (80.0)	0.458 ^#^
	Yes	7 (38.9)	7 (35.0)	4 (20.0)	
Age at onset (years)	Median [IQR]	23.0 [20.0–28.0]	24.0 [21.8–30.0]	NA	0.318 ^§^
Disease duration (years)	Median [IQR]	19.6 [14.7–24.2]	19.2 [10.4–32.7]	NA	0.770 ^§^
History of suicide attempt—N(%)	No	13 (72.2)	17 (85.0)	NA	0.438 ^#^
	Yes	5 (27.8)	3 (15.0)		
Length of treatment with APs (months)	Median [IQR]	66.0 [36.0–165.0]	66.0 [34.0–87.0]	NA	0.348 ^§^
Treatment at sample collection—N(%)	Typical APs (first generation)	3 (16.7)	4 (20.0)	NA	0.261 ^#^
	Atypical APs (second generation)	15 (83.3)	13 (65.0)		
	Aripiprazole (third generation)	0 (0.0)	3 (15.0)		
Mood stabilizers—N(%)	No	11 (61.1)	16 (80.0)	NA	0.288 ^#^
	Yes	7 (38.9)	4 (20.0)		
Antidepressant—N(%)	No	13 (72.2)	14 (70.0)	NA	1.000 ^#^
	Yes	5 (27.8)	6 (30.0)		
Any concomitant drugs—N(%)	No	10 (55.6)	11 (55.0)	NA	1.000 ^#^
	Yes	8 (44.4)	9 (45.0)		

Missing values are excluded, and only valid percentages are reported. * *p*-value from Kruskal–Wallis test; ^§^ *p-*value from Mann–Whitney U test; ^#^ *p*-value from Fisher exact test. Abbreviations: IQR: Interquartile range (i.e., first-third quartiles); NA: not available; APs: antipsychotics; TRS: treatment-resistant schizophrenia; R: treatment-responsive schizophrenia; HC: healthy controls.

**Table 2:** Results from Penalized Logistic Regression Analysis (PELORA) algorithm which identify panels of bacterial populations that best discriminate patients affected by SCZ from HC.

Taxa Level	Bacteria Selected by PELORA	Quantity	Statistics	SCZ (*N* = 38)	HC (*N* = 20)	*p*-Value
Phylum	*Cyanobacteria* [Cluster centroid]	Relative abundance (%)	Median [IQR]	Absent	0.004 [0.002–0.009]	<0.001 ^§^
		Z-score (means)	Mean ± SD	−0.638 ± 0.000	1.211 ± 0.802	
Family	*Paenibacillaceae*	Relative abundance (%)	Median [IQR]	Absent	0.011 [0.006–0.014]	<0.001 ^§^
		Z-score °	Mean ± SD		1.236 ± 0.739	
	*Cytophagaceae*	Relative abundance (%)	Median [IQR]	Absent	0.002 [0.001–0.006]	<0.001 ^§^
		Z-score °	Mean ± SD		1.069 ± 1.079	
	*Morganellaceae*	Relative abundance (%)	Median [IQR]	Absent	0.001 [0.000–0.004]	<0.001 ^§^
		Z-score °	Mean ± SD		0.998 ± 1.183	
	Cluster centroid	Z-score (means)	Mean ± SD	−0.579 ± 0.000	1.101 ± 0.371	<0.001 ^#^
Genus	*Acetanaerobacterium*	Relative abundance (%)	Median [IQR]	Absent	0.004 [0.001–0.015]	<0.001 ^§^
		Z-score °	Mean ± SD		1.124 ± 0.985	
	*Haemophilus*	Relative abundance (%)	Median [IQR]	Absent	0.003 [0.001–0.018]	<0.001 ^§^
		Z-score °	Mean ± SD		1.041 ± 1.122	
	*Turicibacter*	Relative abundance (%)	Median [IQR]	Absent	0.002 [0.000–0.003]	<0.001 ^§^
		Z-score °	Mean ± SD		0.971 ± 1.219	
	*Obesumbacterium*	Relative abundance (%)	Median [IQR]	Absent	0.002 [0.000–0.005]	<0.001 ^§^
		Z-score °	Mean ± SD		0.944 ± 1.253	
	*Gracilibacter*	Relative abundance (%)	Median [IQR]	Absent	0.010 [0.001–0.032]	<0.001 ^§^
		Z-score °	Mean ± SD		1.064 ± 1.087	
	*Intestinibacter*	Relative abundance (%)	Median [IQR]	Absent	0.000 [0.000–0.002]	<0.001 ^§^
		Z-score °	Mean ± SD		0.687 ± 1.498	
	*Hespellia*	Relative abundance (%)	Median [IQR]	Absent	0.006 [0.000–0.017]	<0.001 ^§^
		Z-score °	Mean ± SD		0.974 ± 1.214	
	*Weissella*	Relative abundance (%)	Median [IQR]	Absent	0.001 [0.000–0.001]	<0.001 ^§^
		Z-score °	Mean ± SD		0.872 ± 1.334	
	Cluster centroid	Z-score (means)	Mean ± SD	−0.505 ± 0.000	0.959 ± 0.343	<0.001 ^#^
Species	*Streptococcus equinus*	Relative abundance (%)	Median [IQR]	Absent	0.003 [0.002–0.012]	<0.001 ^§^
		Z-score °	Mean ± SD		1.112 ± 1.007	
	*Coprococcus eutactus*	Relative abundance (%)	Median [IQR]	Absent	0.002 [0.000–0.008]	<0.001 ^§^
		Z-score °	Mean ± SD		0.993 ± 1.190	
	*Turicibacter sanguinis*	Relative abundance (%)	Median [IQR]	Absent	0.002 [0.000–0.003]	<0.001 ^§^
		Z-score °	Mean ± SD		0.965 ± 1.226	
	*Victivallis vadensis*	Relative abundance (%)	Median [IQR]	Absent	0.000 [0.000–0.000]	0.049 ^§^
		Z-score °	Mean ± SD		0.356 ± 1.672	
	*Bariatricus massiliensis*	Relative abundance (%)	Median [IQR]	0.000 [0.000-0.000]	0.000 [0.000–0.001]	0.146 ^§^
		Z-score °	Mean ± SD	−0.087 ± 0.933	0.166 ± 1.123	
	*Prevotella *sp*. Marseille P2931*	Relative abundance (%)	Median [IQR]	Absent	0.000 [0.000–0.000]	0.005 ^§^
		Z-score °	Mean ± SD		0.430 ± 1.644	
	*Faecalitalea cylindroides*	Relative abundance (%)	Median [IQR]	Absent	0.001 [0.000–0.002]	<0.001 ^§^
		Z-score °	Mean ± SD		0.821 ± 1.385	
	*Intestinimonas timonensis*	Relative abundance (%)	Median [IQR]	Absent	0.001 [0.001–0.006]	<0.001 ^§^
		Z-score °	Mean ± SD		1.052 ± 1.106	
	*Bacteroides *sp*. AN 5745*	Relative abundance (%)	Median [IQR]	Absent	0.003 [0.001–0.005]	<0.001 ^§^
		Z-score °	Mean ± SD		1.054 ± 1.103	
	*Collinsella phocaeensis*	Relative abundance (%)	Median [IQR]	Absent	0.000 [0.000–0.000]	<0.001 ^§^
		Z-score °	Mean ± SD		0.626 ± 1.540	
	*Candidatus Dorea massiliensis*	Relative abundance (%)	Median [IQR]	Absent	0.001 [0.000–0.004]	<0.001 ^§^
		Z-score °	Mean ± SD		0.857 ± 1.349	
	*Ruminococcus *sp*. MC 38*	Relative abundance (%)	Median [IQR]	Absent	0.000 [0.000–0.000]	0.049 ^§^
		Z-score °	Mean ± SD		0.293 ± 1.692	
	*Bifidobacterium actinocoloniiforme*	Relative abundance (%)	Median [IQR]	Absent	0.000 [0.000, 0.000]	0.168 ^§^
		Z-score °	Mean ± SD		0.249 ± 1.703	
	*Tidjanibacter massiliensis*	Relative abundance (%)	Median [IQR]	Absent	0.000 [0.000–0.000]	0.001 ^§^
		Z-score °	Mean ± SD		0.478 ± 1.623	
	*Howardella ureilytica*	Relative abundance (%)	Median [IQR]	Absent	0.000 [0.000–0.000]	0.015 ^§^
		Z-score °	Mean ± SD		0.427 ± 1.645	
	*Lactobacillus sanfranciscensis*	Relative abundance (%)	Median [IQR]	Absent	0.000 [0.000–0.000]	0.015 ^§^
		Z-score °	Mean ±SD		0.389 ± 1.660	
	Cluster centroid	Z-score (means)	Mean ± SD	−0.338 ± 0.058	0.642 ± 0.152	<0.001 ^#^

Abbreviations: IQR: Interquartile range (i.e., first-third quartiles); SD: Standard Deviation; SCZ: schizophrenia; HC: healthy controls. Absent: all values are 0%. ° Standardized Z-score: the relative abundance of each bacterium was first logistic transformed and then the Z-score was calculated subtracting its mean and dividing by its standard deviation (SD). Both the mean and SD were computed in the sample which included all SCZ and HC. Centroid is computed by the mean of Z-scores; ^#^ *p*-values from two-sample *t*-test on Z-scores; ^§^ *p*-values from Mann-Whitney U test, calculated in presence of no variance in one of the two groups. Background color it indicates the centroid.

**Table 3:** Results from Penalized Logistic Regression Analysis (PELORA) algorithm which identifies panels of bacterial populations that best discriminate TRS and R patients.

Taxa Level	Bacteria Selected by PELORA	Quantity	Statistics	TRS (*N* = 18)	R (*N* = 20)	*p*-Value
Phylum	*Candidatus Saccharibacteria*	Relative abundance (%)	Median [IQR]	0.006 [0.005-0.012]	Absent	<0.001 ^§^
		Z-score °	Mean ± SD	0.940 ± 0.632		
	*Tenericutes*	Relative abundance (%)	Median [IQR]	0.003 [0.001–0.005]	Absent	<0.001 ^§^
		Z-score °	Mean ± SD	0.686 ± 1.109		
	Cluster centroid	Z-score (means)	Mean ± SD	0.813 ± 0.463	−0.732 ± 0.000	<0.001 ^#^
Family	*Flavobacteriaceae*	Relative abundance (%)	Median [IQR]	Absent	0.005 [0.003–0.009]	<0.001 ^§^
		Z-score °	Mean ± SD		0.830 ± 0.646	
	*Enterococcaceae*	Relative abundance (%)	Median [IQR]	Absent	0.003 [0.001–0.010]	<0.001 ^§^
		Z-score °	Mean ± SD		0.692 ± 0.940	
	Cluster centroid	Z-score (means)	Mean ± SD	−0.845 ± 0.000	0.761 ± 0.512	<0.001 ^#^
Genus	*Actinomyces*	Relative abundance (%)	Median [IQR]	0.005 [0.002–0.016]	Absent	<0.001 ^§^
		Z-score °	Mean ± SD	0.906 ± 0.724		
	*Porphyromonas*	Relative abundance (%)	Median [IQR]	0.003 [0.001–0.008]	Absent	<0.001 ^§^
		Z-score °	Mean ± SD	0.772 ± 0.988		
	Cluster centroid	Z-score (means)	Mean ± SD	0.839 ± 0.483	−0.755 ± 0.000	<0.001 ^#^
Species	Flintibacter butyricus [cluster centroid]	Relative abundance (%)	Median [IQR]	Absent	0.017 [0.008–0.031]	<0.001 ^§^
		Z-score °	Mean ± SD	−0.989 ± 0.000	0.890 ± 0.430	

Abbreviations: IQR: Interquartile range (i.e., first-third quartiles); SD: Standard Deviation; SCZ: schizophrenia; TRS: treatment-resistant schizophrenia; R: treatment-responsive; HC: healthy controls. Absent: all values are 0%. ° Standardized Z-score: the relative abundance of each bacterium was first logistic transformed and then the Z-score was calculated subtracting its mean and dividing by its standard deviation (SD). Both the mean and SD were computed in the sample which included all SCZ and HC. Centroid is computed by the mean of Z-scores; ^#^ *p*-values from two-sample *t*-test on Z-scores; ^§^ *p*-values from Mann–Whitney U test, calculated in presence of no variance in one of the two groups. Background color it indicates the centroid.

**Table 4:** Results from Penalized Logistic Regression Analysis (PELORA) algorithm which identifies panels of bacterial populations that best discriminate R patients with SCZ from HC.

Taxa Level	Bacteria Selected by PELORA	Quantity	Statistics	R (*N* = 20)	HC (*N* = 20)	*p*-Value
Phylum	*Candidatus Saccharibacteria*	Relative abundance (%)	Median [IQR]	Absent	0.006 [0.004–0.014]	<0.001 ^§^
		Z-score °	Mean ± SD		0.869 ± 0.681	
	*Cyanobacteria*	Relative abundance (%)	Median [IQR]	Absent	0.004 [0.002–0.009]	<0.001 ^§^
		Z-score °	Mean ± SD		0.848 ± 0.735	
	*Tenericutes*	Relative abundance (%)	Median [IQR]	Absent	0.024 [0.003–0.111]	<0.001 ^§^
		Z-score °	Mean ± SD		0.825 ± 0.787	
	Cluster centroid	Z-score (means)	Mean ± SD	−0.847 ± 0.000	0.847 ± 0.415	<0.001 ^#^
Family	*Flavobacteriaceae*	Relative abundance (%)	Median [IQR]	0.005 [0.003–0.009]	Absent	<0.001 ^§^
		Z-score °	Mean ± SD	0.880 ± 0.650		
	*Desulfobacteraceae*	Relative abundance (%)	Median [IQR]	0.000 [0.000-0.003]	Absent	<0.001 ^§^
		Z-score °	Mean ± SD	0.462 ± 1.266		
	Cluster centroid	Z-score (means)	Mean ± SD	0.671 ± 0.675	−0.671 ± 0.000	<0.001 ^#^
Genus	*Fenollaria*	Relative abundance (%)	Median [IQR]	0.007 [0.004–0.012]	Absent	<0.001 ^§^
		Z-score °	Mean ± SD	0.845 ± 0.740		
	*Mitsuokella*	Relative abundance (%)	Median [IQR]	0.000 [0.000–0.002]	Absent	0.002 ^§^
		Z-score °	Mean ± SD	0.432 ± 1.288		
	*Harryflintia*	Relative abundance (%)	Median [IQR]	0.001 [0.000–0.007]	Absent	<0.001 ^§^
		Z-score °	Mean ± SD	0.573 ± 1.167		
	*Mailhella*	Relative abundance (%)	Median [IQR]	0.000 [0.000–0.002]	Absent	0.002 ^§^
		Z-score °	Mean ± SD	0.432 ± 1.289		
	Cluster centroid	Z-score (means)	Mean ± SD	0.570 ± 0.341	−0.570 ± 0.000	<0.001 ^#^
Species	Flintibacter butyricus [cluster centroid]	Relative abundance (%)	Median [IQR]	0.017 [0.008–0.031]	Absent	<0.001 ^#^
		Z-score °	Mean ± SD	0.942 ± 0.431	−0.942 ± 0.000	

Abbreviations: IQR: Interquartile range (i.e., first-third quartiles); SD: Standard Deviation; R: treatment-responsive schizophrenia; HC: healthy controls. Absent: all values are 0%. ° Standardized Z-score: the relative abundance of each bacterium was first logistic transformed and then the Z-score was calculated subtracting its mean and dividing by its standard deviation (SD). Both the mean and SD were computed in the sample which included all patients affected by SCZ and HC. Centroid is computed by the mean of Z-scores; ^#^ *p*-values from two-sample *t*-test on Z-scores; ^§^ *p*-values from Mann–Whitney U test, calculated in presence of no variance in one of the two groups. Background color it indicates the centroid.

**Table 5:** Results from Penalized Logistic Regression Analysis (PELORA) algorithm which identifies panels of bacterial populations that best discriminate TRS from HC.

Taxa Level	Bacteria Selected by PELORA	Quantity	Statistics	TRS (*N* = 18)	HC (*N* = 20)	*p*-Value
Phylum	*Cyanobacteria* [cluster centroid]	Relative abundance (%)	Median [IQR]	Absent	0.004 [0.002–0.009]	<0.001 ^§^
		Z-score °	Mean ± SD	−0.886 ± 0.000	0.798 ± 0.730	
Family	*Enterococcaceae*	Relative abundance (%)	Median [IQR]	Absent	0.007 [0.002–0.012]	<0.001 ^§^
		Z-score °	Mean ± SD		0.744 ± 0.847	
	*Paenibacillaceae*	Relative abundance (%)	Median [IQR]	Absent	0.011 [0.006–0.014]	<0.001 ^§^
		Z-score °	Mean ± SD		0.819 ± 0.677	
	*Cytophagaceae*	Relative abundance (%)	Median [IQR]	Absent	0.002 [0.001–0.006]	<0.001 ^§^
		Z-score °	Mean ± SD		0.683 ± 0.954	
	*Hafniaceae*	Relative abundance (%)	Median [IQR]	Absent	0.003 [0.001–0.010]	<0.001 ^§^
		Z-score °	Mean ± SD		0.660 ± 0.989	
	*Pasteurellaceae*	Relative abundance (%)	Median [IQR]	Absent	0.006 [0.003–0.023]	<0.001 ^§^
		Z-score °	Mean ± SD		0.745 ± 0.844	
	Cluster centroid	Z-score (means)	Mean ± SD	−0.811 ± 0.000	0.730 ± 0.320	<0.001 ^#^
Genus	*Murimonas*	Relative abundance (%)	Median [IQR]	Absent	0.019 [0.008–0.037]	<0.001 ^§^
		Z-score °	Mean ± SD		0.849 ± 0.587	
	*Haemophilus*	Relative abundance (%)	Median [IQR]	Absent	0.003 [0.001–0.018]	<0.001 ^§^
		Z-score °	Mean ± SD		0.662 ± 0.987	
	*Peptococcus*	Relative abundance (%)	Median [IQR]	Absent	0.006 [0.000–0.010]	<0.001 ^§^
		Z-score °	Mean ± SD		0.615 ± 1.052	
	*Weissella*	Relative abundance (%)	Median [IQR]	Absent	0.001 [0.000–0.001]	<0.001 ^§^
		Z-score °	Mean ± SD		0.539 ± 1.141	
	*Enterobacter*	Relative abundance (%)	Median [IQR]	Absent	0.001 [0.000–0.040]	<0.001 ^§^
		Z-score °	Mean ± SD		0.538 ± 1.142	
	*Hespellia*	Relative abundance (%)	Median [IQR]	Absent	0.006 [0.000–0.017]	<0.001 ^§^
		Z-score °	Mean ± SD		0.612 ± 1.056	
	*Turicibacter*	Relative abundance (%)	Median [IQR]	Absent	0.002 [0.000–0.003]	<0.001 ^§^
		Z-score °	Mean ± SD		0.610 ± 1.059	
	*Fusicatenibacter*	Relative abundance (%)	Median [IQR]	0.005 [0.003–0.018]	0.010 [0.005–0.038]	0.334 ^#^
		Z-score °	Mean ± SD	−0.167 ± 1.062	0.151 ± 0.942	
	*Eggerthella*	Relative abundance (%)	Median [IQR]	0.013 [0.007–0.079]	0.017 [0.010–0.043]	0.920 ^#^
		Z-score °	Mean ± SD	0.018 ± 1.024	−0.016 ± 1.004	
	Cluster centroid	Z-score (means)	Mean ± SD	−0.563 ± 0.125	0.507 ± 0.168	<0.001 ^#^
Species	*Bacteroides *sp*. Marseille P3108*	Relative abundance (%)	Median [IQR]	0.005 [0.003–0.016]	Absent	<0.001 ^§^
		Z-score °	Mean ± SD	0.888 ± 0.767		
	*Pseudoflavonifractor capillosus*	Relative abundance (%)	Median [IQR]	0.009 [0.002–0.028]	Absent	<0.001 ^§^
		Z-score °	Mean ± SD	0.834 ± 0.881		
	*Erysipelatoclostridium ramosum*	Relative abundance (%)	Median [IQR]	0.001 [0.000–0.019]	Absent	<0.001 ^§^
		Z-score °	Mean ± SD	0.613 ± 1.192		
	*Papillibacter cinnamivorans*	Relative abundance (%)	Median [IQR]	0.006 [0.000–0.018]	Absent	<0.001 ^§^
		Z-score °	Mean ± SD	0.735 ± 1.044		
	*Clostridium *sp*. BPY5*	Relative abundance (%)	Median [IQR]	0.007 [0.001–0.016]	Absent	<0.001 ^§^
		Z-score °	Mean ± SD	0.784 ± 0.969		
	Cluster centroid	Z-score (means)	Mean ± SD	0.771 ± 0.204	−0.694 ± 0.000	<0.001 ^#^

Abbreviations: IQR: Interquartile range (i.e., first-third quartiles); SD: Standard Deviation; SCZ: schizophrenia; TRS: treatment-resistant schizophrenia; HC: healthy controls Absent: all values are 0%. ° Standardized Z-score: the relative abundance of each bacterium was first logistic transformed and then the Z-score was calculated subtracting its mean and dividing by its standard deviation (SD). Both the mean and SD were computed in the sample which included all patients affected by SCZ and HC. Centroid is computed by the mean of Z-scores; ^#^ *p*-values from two-sample *t*-test on Z-scores; ^§^ *p*-values from Mann–Whitney U test, calculated in presence of no variance in one of the two groups. Background color it indicates the centroid.

**Table 6:** Results from Penalized Logistic Regression Analysis (PELORA) algorithm which identifies panels of bacterial populations that best discriminate patients affected by SCZ treated with typical versus atypical antipsychotics (including aripiprazole).

Taxa Level	Bacteria Selected by PELORA	Quantity	Statistics	T SCZ (*N* = 7)	AT SCZ (*N* = 31)	*p*-Value
Phylum	*Fusobacteria*	Relative abundance (%)	Median [IQR]	0.012 [0.001-0.039]	0.000 [0.000–0.001]	0.001 ^#^
		Z-score °	Mean ± SD	1.103 ± 1.223	−0.249 ± 0.766	
	Tenericutes *	Relative abundance (%)	Median [IQR]	0.000 [0.000–0.000]	0.000 [0.000–0.003]	0.153 ^#^
		Z-score °	Mean ± SD	−0.491 ± 0.335	0.111 ± 1.069	
	Bacteroidetes *	Relative abundance (%)	Median [IQR]	42.397 [26.873–44.399]	37.181 [28.697–49.898]	0.543 ^#^
		Z-score °	Mean ± SD	−0.211 ± 0.951	0.048 ± 1.020	
	Cluster centroid	Z-score (means)	Mean ± SD	0.602 ± 0.314	–0.136 ± 0.434	<0.001 ^#^
Family	*Fusobacteriaceae*	Relative abundance (%)	Median [IQR]	0.012 [0.000–0.039]	0.000 [0.000–0.001]	0.001 ^#^
		Z-score °	Mean ± SD	1.036 ± 1.351	−0.234 ± 0.749	
	*Streptomycetaceae*	Relative abundance (%)	Median [IQR]	0.011 [0.009–0.015]	0.008 [0.004–0.018]	0.438 ^#^
		Z-score °	Mean ± SD	0.269 ± 0.876	−0.061 ± 1.029	
	*Helicobacteraceae*	Relative abundance (%)	Median [IQR]	0.001 [0.000–0.094]	0.000 [0.000–0.002]	0.085 ^#^
		Z-score °	Mean ± SD	0.588 ± 1.609	−0.133 ± 0.785	
	Clostridiales Family XII. Incertae Sedis *	Relative abundance (%)	Median [IQR]	Absent	0.000 [0.000–0.001]	0.070 ^§^
		Z-score °	Mean ± SD		0.125 ± 1.071	
	*Bacillaceae*	Relative abundance (%)	Median [IQR]	0.012 [0.011–0.017]	0.007 [0.005–0.013]	0.122 ^#^
		Z-score °	Mean ± SD	0.530 ± 0.798	−0.120 ± 1.013	
	*Oxalobacteraceae*	Relative abundance (%)	Median [IQR]	0.008 [0.002–0.020]	0.002 [0.000–0.018]	0.366 ^#^
		Z-score °	Mean ± SD	0.314 ± 0.635	−0.071 ± 1.060	
	Synergistaceae *	Relative abundance (%)	Median [IQR]	0.001 [0.000–0.013]	0.002 [0.000–0.006]	0.603 ^#^
		Z-score °	Mean ± SD	−0.181 ± 0.995	0.041 ± 1.013	
	*Erysipelotrichaceae*	Relative abundance (%)	Median [IQR]	0.138 [0.090–1.438]	0.092 [0.046–0.450]	0.338 ^#^
		Z-score °	Mean ± SD	0.332 ± 1.111	−0.075 ± 0.977	
	Clostridiales Family XIII Incertae Sedis *	Relative abundance (%)	Median [IQR]	0.006 [0.000–0.017]	0.013 [0.002–0.022]	0.107 ^#^
		Z-score °	Mean ± SD	−0.551 ± 1.302	0.124 ± 0.898	
	Veillonellaceae *	Relative abundance (%)	Median [IQR]	0.146 [0.044–1.892]	0.836 [0.125–3.110]	0.590 ^#^
		Z-score °	Mean ± SD	−0.188 ± 1.189	0.042 ± 0.970	
	Cluster centroid	Z-score (means)	Mean ± SD	0.454 ± 0.159	–0.103 ± 0.147	<0.001 ^#^
Genus	*Fusobacterium*	Relative abundance (%)	Median [IQR]	0.012 [0.000–0.039]	0.000 [0.000–0.000]	0.001 ^#^
		Z-score °	Mean ± SD	1.067 ± 1.351	−0.241 ± 0.736	
	*Butyricimonas*	Relative abundance (%)	Median [IQR]	0.800 [0.341–0.895]	0.229 [0.103–0.484]	0.201 ^#^
		Z-score °	Mean ± SD	0.440 ± 0.751	−0.099 ± 1.032	
	*Blautia*	Relative abundance (%)	Median [IQR]	1.794 [0.639–6.945]	0.544 [0.323–0.831]	0.018 ^#^
		Z-score °	Mean ± SD	0.791 ± 1.066	−0.179 ± 0.909	
	*Paraprevotella*	Relative abundance (%)	Median [IQR]	0.053 [0.007–1.231]	0.007 [0.002–0.562]	0.395 ^#^
		Z-score °	Mean ± SD	0.295 ± 1.130	−0.067 ± 0.976	
	Klebsiella *	Relative abundance (%)	Median [IQR]	0.001 [0.000–0.003]	0.000 [0.000–0.003]	0.889 ^#^
		Z-score °	Mean ± SD	−0.049 ± 0.674	0.011 ± 1.069	
	Olsenella *	Relative abundance (%)	Median [IQR]	0.000 [0.000–0.000]	0.000 [0.000–0.000]	0.735 ^#^
		Z-score °	Mean ± SD	−0.118 ± 0.944	0.027 ± 1.025	
	*Cronobacter*	Relative abundance (%)	Median [IQR]	0.000 [0.000–0.000]	0.000 [0.000–0.000]	0.142 ^#^
		Z-score °	Mean ± SD	0.504 ± 1.783	−0.114 ± 0.725	
	Cluster centroid	Z-score (means)	Mean ± SD	0.466 ± 0.264	–0.105 ± 0.156	<0.001 ^#^
Species	*Parabacteroides merdae*	Relative abundance (%)	Median [IQR]	0.000 [0.000–0.001]	0.000 [0.000–0.000]	0.006 ^#^
		Z-score °	Mean ± SD	0.910 ± 2.108	−0.205 ± 0.329	
	*Parabacteroides *sp*. DJF B084*	Relative abundance (%)	Median [IQR]	0.000 [0.000–0.109]	0.000 [0.000–0.000]	0.025 ^#^
		Z-score °	Mean ± SD	0.753 ± 1.805	−0.170 ± 0.648	
	*Collinsella massiliensis*	Relative abundance (%)	Median [IQR]	0.000 [0.000–0.000]	0.000 [0.000–0.000]	0.069 ^#^
		Z-score °	Mean ± SD	0.620 ± 2.198	−0.140 ± 0.397	
	*Odoribacter *sp*. S90*	Relative abundance (%)	Median [IQR]	0.000 [0.000–0.000]	Absent	0.035 0.035 ^§^
		Z-score °	Mean ± SD	0.718 ± 2.330		
	*Ruminococcus *sp*. AT10*	Relative abundance (%)	Median [IQR]	0.000 [0.000–0.003]	0.000 [0.000–0.000]	0.132 ^#^
		Z-score °	Mean ± SD	0.517 ± 1.828	−0.117 ± 0.699	
	*Clostridium piliforme*	Relative abundance (%)	Median [IQR]	0.000 [0.000–0.002]	0.000 [0.000–0.000]	0.051 ^#^
		Z-score °	Mean ± SD	0.663 ± 1.848	−0.150 ± 0.651	
	*Enorma massiliensis*	Relative abundance (%)	Median [IQR]	0.000 [0.000–0.091]	0.000 [0.000–0.000]	0.109 ^#^
		Z-score °	Mean ± SD	0.549 ± 1.632	−0.124 ± 0.784	
	*Megamonas rupellensis*	Relative abundance (%)	Median [IQR]	0.000 [0.000–0.187]	0.000 [0.000–0.000]	0.061 ^#^
		Z-score °	Mean ± SD	0.637 ± 1.703	−0.144 ± 0.733	
	*Bacteroides ovatus*	Relative abundance (%)	Median [IQR]	0.902 [0.736–1.158]	0.644 [0.004–1.191]	0.077 ^#^
		Z-score °	Mean ± SD	0.604 ± 0.351	−0.136 ± 1.051	
	*Butyricimonas *sp*. GD2*	Relative abundance (%)	Median [IQR]	0.042 [0.000–0.185]	0.000 [0.000–0.068]	0.352 ^#^
		Z-score °	Mean ± SD	0.322 ± 1.170	−0.073 ± 0.964	
	Faecalibacterium prausnitzii *	Relative abundance (%)	Median [IQR]	0.920 [0.450–2.506]	5.561 [2.425–9.540]	0.061 ^#^
		Z-score °	Mean ± SD	−0.636 ± 0.900	0.144 ± 0.978	
	*Ruminococcus *sp*. DJF VR70k1*	Relative abundance (%)	Median [IQR]	0.000 [0.000–0.004]	0.000 [0.000–0.003]	0.910 ^#^
		Z-score °	Mean ± SD	0.039 ± 0.861	−0.009 ± 1.042	
	Clostridium aldenense *	Relative abundance (%)	Median [IQR]	0.006 [0.006–0.118]	0.172 [0.035–0.495]	0.017 ^#^
		Z-score °	Mean ± SD	−0.802 ± 1.265	0.181 ± 0.854	
	Tetragenococcus halophilus *	Relative abundance (%)	Median [IQR]	Absent	0.000 [0.000–0.000]	0.322 ^§^
		Z-score °	Mean ± SD		0.064 ± 1.100	
	Cluster centroid	Z-score (means)	Mean ± SD	0.575 ± 0.093	−0.130 ± 0.074	<0.001 ^#^

Abbreviations: IQR: Interquartile range (i.e., first-third quartiles); SD: Standard Deviation; SCZ: schizophrenia, T: typical antipsychotics; AT: atypical antipsychotics. Absent: all values are 0%. ° Standardized Z-score: the relative abundance of each bacterium was first logistic transformed and then the Z-score was calculated subtracting its mean and dividing by its standard deviation (SD). Both the mean and SD were computed in the sample which included all patients affected by SCZs and HC. Centroid is computed by the mean of Z-scores; * to calculate the centroid, the sign of the specific bacterium’s Z-score was reversed; ^#^ *p*-values from two-sample *t*-test on Z-scores; ^§^ *p*-values from Mann–Whitney U test, calculated in presence of no variance in one of the two groups. Background color it indicates the centroid.

## Data Availability

The data presented in this study are available on request from the corresponding author. The data are not publicly available.
